# A Constitutional Amendment for Deworming

**DOI:** 10.1371/journal.pntd.0000454

**Published:** 2009-08-25

**Authors:** Peter J. Hotez

**Affiliations:** 1 Department of Microbiology, Immunology, and Tropical Medicine, The George Washington University, Washington, D.C., United States of America; 2 Sabin Vaccine Institute, Washington, D.C., United States of America

*“Canek said: every human being, by being a human, has attributes that are expressions of his essence and voices that reveal his origin and condition. The attribute of the ocean is pride; the attribute of the sun is authority; the attribute of a human is dignity.”*

*E. Abreu Gómez, 1969 [1]*

In February 2009, *The Washington Post* reported that three South American countries, Bolivia, Ecuador, and Venezuela, have either launched or completed ambitious efforts to rewrite their constitutions in order to expand the social and economic rights guaranteed to its citizens [2]. For the most part these new charters are considered populist documents, reflecting the leanings of newly elected leftist regimes and emphasizing the rights of poor and indigenous people, i.e., populations that have suffered decades or even centuries of neglect in previous regimes. In Venezuela, Hugo Chavez has ruled the longest—a decade—while both Bolivia's Evo Morales (who became the region's first indigenous leader) and Ecuador's Rafael Correa campaigned and won elections based on their pledges to reverse centuries of discrimination [3].

The new populist constitutions have been described as “sprawling” [2]. Written with the help of a team of Spanish legal scholars led by Roberto Viciano Pastor, the documents list as many as 444 rights, including guarantees to food, water, public health, and lives free of torture [2]. The rights of children and women, especially those from indigenous backgrounds, receive special attention, and in some cases include freedom from physical and sexual violence [2].

In past articles and editorials published in *PLoS Neglected Tropical Diseases* I have emphasized the vulnerability of children and women living in the Latin American and Caribbean region, particularly those of indigenous and African descent, to the neglected tropical diseases (NTDs) [4]–[6]. As the most common conditions affecting the poorest 100 million people in Latin America and the Caribbean, the NTDs represent major reasons why the poor face a lifetime of discrimination, isolation, ill health, poverty, and overall deprivation [4],[7]. In Bolivia, Ecuador, and Venezuela, the most common NTDs are the three major intestinal helminth infections, trichuriasis, ascariasis, and hookworm infection [4],[8]. Shown in Table 1 are the number of cases and prevalence of these helminthiases, with up to 15% of Bolivia's population infected, 38% of Ecuador's population, and 35% of Venezuela's population [8]. What does it mean that millions of indigenous people in these countries harbor ascaris roundworms, trichuris whipworms, and necator hookworms in their intestines? Children usually have the largest numbers of these parasites and as a result they suffer from deficits in physical growth, as well as reductions in intelligence, memory, and cognition [9],[10]. On this basis, the intestinal helminths have been shown to not only impair health, but also education, which in turn translates into reduced future wage earnings [10],[11]. Thus, the intestinal worms are important reasons why children will never grow to their full potential and why they may never escape poverty [7],[10],[11]. In the Americas, young women and pregnant women also endure high rates of hookworm infection, and as a result they are at risk for increased morbidity and mortality and for giving birth to children with low birth weights and reduced survival [4],[12].

**Table 1:** Number of Cases and Prevalence of the Major Intestinal Helminth Infections in Bolivia, Ecuador, and Venezuela.

Country (Population)	Ascariasis	Trichuriasis	Hookworm Infection
	Number of Cases/Prevalence	Number of Cases/Prevalence	Number of Cases/Prevalence
Bolivia (9 million)	1.3 million/15%	065 million/7%	1.3 million/15%
Ecuador (13 million)	4.9 million38%	1.6 million/12.5%	0.8 million/6%
Venezuela (25 million)	7.4 million/29%	8.7 million/35%	1.6 million/6%

Data are from [7].

The fact that millions of indigenous children and women in Latin America suffer from intestinal helminth infections is especially tragic because we can do something about these conditions through interventions that are astonishingly low cost [4]. In many cases, once-yearly deworming with a benimidazole anthelminthic drug such as albendazole or mebendazole is sufficient [13],[14]. These drugs are particularly effective for treating ascaris and trichuris worm infections [14],[15] and can be administered for as little as US$0.03 per person [16],[17]. For me it is shocking to think that we could allow vulnerable children and women to live with chronic intestinal helminth infections when such NTDs can be controlled through mass drug administration for just a few pennies per individual. Even if we consider all of the roughly 50 million people living in Bolivia, Ecuador, and Venezuela, the costs of annual deworming in national programs of control could be accomplished for less than US$5–10 million. Given the rapid impact and cost-effectiveness of deworming in terms of improving child development and education, pregnancy outcome, and ultimately poverty reduction, it is shameful that we allow this situation to continue. Globally it is estimated that only about 10% of school-aged children and 20% of preschool children who are at risk for acquiring intestinal helminth infections receive anthelminthic drugs [13],[14].

In order to address this health disparity in the Latin American and Caribbean region, the Global Network for Neglected Tropical Diseases together with the Interamerican Development Bank, the Pan American Health Organization, and the World Health Organization have recently established a program to ensure that vulnerable populations living in the region have access to essential medicines for NTDs, including benzimidazole anthelminthics [7],[18]. In the Andean region, where many of the indigenous people of Bolivia and Ecuador live, additional interventions may also include treatments and vector control programs for Chagas disease, cysticercosis, echinococcosis, fascioliasis, leishmaniasis, and other condition (Box 1). Simultaneously, and given the health and social benefits of deworming, it may also make sense to guarantee access to essential anthelminthic drugs along with other basic human rights related to maternal and child public health. A constitutional amendment for deworming would announce a governmental commitment to address one of the most glaring health disparities for indigenous children and women living in Bolivia, Ecuador, and Venezuela. Together with co-investments from the Global Network for Neglected Tropical Diseases, deworming would represent an extremely modest yet highly cost-effective expenditure for improving education, reducing poverty, and ending centuries of neglect.

**Box 1. Major Neglected Tropical Diseases in the Andean Region**

Ascariasis

Bartonellosis

Chagas disease

Cysticercosis

Ectoparasites

Echinocossis

Fascioliasis

Hookworm infection

Strongyloidiasis

Trichuriasis

Leishmaniasis

Plague

Adapted from [3].

Amending the constitution to provide national commitments to deworming could also stimulate other governments in the region to launch or expand NTD control campaigns. It has been estimated that today approximately 75% of South America's 382 million inhabitants live under leftist governments, including the governments of Argentina, Brazil, Chile, and Uruguay as well as the Andean countries mentioned above [3]. National and regional investments in deworming would help to redress centuries of discrimination. Moreover, success on this front might also lead to investments for research and development into additional and improved control tools, including a new generation of so-called “antipoverty” vaccines to combat hookworm infection, leishmaniasis, schistosomiasis, and other NTDs [19]. I have further suggested that the pending closure of the Guantanamo military base could give way to a replacement facility that includes an international tropical disease research institute devoted to such activities [20]. The new constitutions and the Global Network for Neglected Tropical Diseases may promote important new changes for the health and education of the poorest people in Latin America and elsewhere. Given the extremely modest costs, the rapid health impact, and the resulting high rates of economic return, we cannot afford further delays in implementing large-scale NTD control measures.
